# Reduction of Arterial Stiffness by Exercise Training Is Associated with Increasing Plasma Apelin Level in Middle-Aged and Older Adults

**DOI:** 10.1371/journal.pone.0093545

**Published:** 2014-04-01

**Authors:** Shumpei Fujie, Koji Sato, Eri Miyamoto-Mikami, Natsuki Hasegawa, Satoshi Fujita, Kiyoshi Sanada, Takafumi Hamaoka, Motoyuki Iemitsu

**Affiliations:** Faculty of Sport and Health Science, Ritsumeikan University, Shiga, Japan; University of Alabama, Birmingham, United States of America

## Abstract

Aging-induced deterioration of arterial stiffness is decreased by regular exercise, and increased nitric oxide (NO) production participates in this effect. Apelin regulates endothelial NO synthase in endothelial cells, promoting NO production. However, the effect of aerobic exercise training on circulating apelin levels in healthy middle-aged and older adults remains unknown. Accordingly, this study aimed to clarify the effects of regular aerobic exercise on apelin concentrations in middle-aged and older adults. Thirty-four healthy middle-aged and older subjects (67.0 ± 1.3 years) were randomly divided into two groups: exercise intervention and sedentary controls. Subjects in the training group completed 8-week of aerobic exercise training (60–70% peak oxygen uptake [VO_2peak_] for 45 min, 3 days/week). Before and after the intervention, we evaluated plasma apelin and nitrite/nitrate (NOx) concentrations, VO_2peak_, and arterial stiffness index. In the training group, VO_2peak_ was significantly increased, and carotid β-stiffness was significantly decreased, after the intervention (*P*<0.05). Moreover, plasma apelin and NOx levels were significantly increased in the training group after the intervention (*P*<0.05). Additionally, there was a correlation between the training effects of plasma apelin levels and carotidβ-stiffness (r = −0.508, *P* = 0.032) and plasma NOx levels (r = 0.494, *P* = 0.037). By contrast, none of these parameters changed significantly in the control group. These results suggest that the increased in plasma apelin levels may be associated with exercise training-induced alternation of arterial stiffness in middle-aged and older adults.

## Introduction

Alterations in arterial structure and function occur with advancing age in healthy individuals [1], and the aging-induced decrease in endothelial function contributes to the increases in arterial stiffness [2]–[5]. Several studies have shown that arterial stiffness is lower in physically active individuals compared with sedentary individuals [1], [6], [7]. Furthermore, aerobic exercise training reduces arterial stiffness, which increases with advancing age [7], [8]. Thus, regular aerobic exercise prevents or reduces arterial stiffness. Nitric oxide (NO), which is produced from L-arginine by endothelial NO synthase (eNOS) in endothelial cells, contributes to the underlying mechanism of this effect of exercise. NO causes vasodilation and inhibits the development of arteriosclerosis and atherosclerosis [9]. Aging impairs arterial eNOS protein and mRNA expression, however, eNOS expression levels are increased by endurance exercise training in aged rats [10]. Moreover, in middle-aged and older woman, moderate regular exercise training elevates plasma NOx levels with reduction of blood pressure [11]. Thus, aging impairs NO bioavailability, and it may result in increased arterial stiffness.

Recently, apelin has been detected in several tissues, such as white adipose tissue, kidney, heart, and vessel [12], [13]. Apelin is initially synthesized as preproapelin, which consists of 77 amino-acid residues. Following enzymatic cleavage, the C-terminus is released into the circulation as the biologically active fragment, apelin [14]. Apelin acts via APJ receptor in the expressing endothelial cells [12], [13]. Clinical evidence suggests that plasma apelin levels are generally lower in patients with cardiovascular diseases, such as heart failure and hypertension [15], [16]. In animal studies, apelin administration decreased blood pressure in normal and hypertensive rats [17]–[19]. This effect was blocked in the presence of a NOS inhibitor, suggesting that apelin causes vasodilation through a mechanism that involves NO [17]. In the APJ-deficient mice, the hypotensive effect to apelin was blocked with the downregulating eNOS phosphorylation in the endothelial cells [17]. Therefore, reduced plasma apelin levels may be associated with reductions of NO bioavailability in older adults, and this may in turn result in increased arterial stiffness. Additionally, in patients with type 2 diabetes mellitus, regular exercise training elevates circulating apelin levels, and higher levels of physical activity caused larger increases in apelin levels than lower levels of activity [20], [21]. Furthermore, a study in hypertensive rats showed that exercise training promotes mRNA expression and tissue concentration of apelin in the aorta [22]. However, the association between aerobic exercise training-induced changes in arterial stiffness and circulating apelin level in healthy middle-aged and older adults remains unclear.

We hypothesized that aerobic exercise training would elevate plasma apelin levels along with plasma NOx levels in middle-aged and older adults, and that this increase in apelin may be participated in endurance exercise training-induced reduction of arterial stiffness. To test our hypothesis, we measured plasma apelin levels, nitrite/nitrate (NOx) concentrations, and arterial stiffness in middle-aged and older adults using a randomized controlled exercise intervention trial.

## Methods

### Subjects

Thirty-four healthy middle-aged and older subjects (total, *n* = 34, 67.0±1.3 years; male, *n* = 14, 70.4±1.6 years; female, *n* = 20, 64.7±1.8 years) volunteered to participate in this study. Subjects were recruited via advertisement from a local community health center and a community recreation center. All volunteers provided written informed consent before participating in the study, which was approved by the Ethics Committee of Ritsumeikan University and was conducted in accordance with the Declaration of Helsinki. Subjects with taking medication such as anti-hyperlipidemic, anti-hypertensive, or anti-hyperglycemic, and a history of stroke, diabetes, hypertension, hyperlipidemia, cardiac disease, chronic renal failure, and mental disorder were excluded from the study, and the subjects in this study hardly drank alcohol. Subjects were randomly divided into two groups: the training group (*n* = 18 [male  =  7/female  = 11], 66.4±2.1 years) and the control group (*n* = 16 [male  =  7/female  =  9], 67.8±1.5 years).

### Experimental design

For all subjects, VO_2 peak_, body weight, body fat, height, resting systolic blood pressure (SBP), resting diastolic blood pressure (DBP), resting heart rate (HR), resting plasma NOx concentrations, resting plasma apelin concentrations, and serum concentrations of total cholesterol, HDL cholesterol, and triglycerides were measured at the beginning and end of the experiment. Carotidβ-stiffness was examined as an index of arterial stiffness. Before subjects were tested, they sat quietly for 30 min. Resting brachial SBP, DBP, and HR were measured in duplicate in the supine position at rest, using a vascular testing device (OMRON COLIN Co., Tokyo, Japan). At the beginning and end of the study period, fasting blood samples were drawn following at least 48 hours of rest after the last exercise-training session. All subjects were instructed not to eat or drink fluids other than water for at least 12 h prior to blood sampling. In addition, we checked to be sure that participants did not intake any dietary sources of NOx over the 24 h prior to testing in both groups, since NOx can be affected by diet. Thus we were able to rule out both acute effects from the most recent bout of exercise and oral sources of NOx other than NO. Serum and plasma samples were immediately centrifuged (1500×*g*, 15 min, 4°C). Blood samples were stored at −80°C until use. Room temperature was maintained at 24°C throughout the experiment.

### Exercise intervention

Aerobic exercise-training program consisted of cycling on a leg ergometer (828E Monark cycle ergometer, Stockholm, Sweden) for 55 min, 3 days/week, for 8 weeks. Each exercise session consisted of a 5-min warm-up period at 40% peak oxygen uptake (VO_2peak_), followed by 45 min of cycling at a resistance that elicited 60–70% VO_2peak_, and ended with a 5-min cool-down period at 40% VO_2peak_. The exercise training program was conducted in the group at AM9:00–11:00 after breakfast. Exercise compliance was carefully monitored by direct supervision. Additionally, the sedentary control subjects were encouraged to maintain the activities of daily living and might not be changed during the 8-week experiment period. The subjects in both groups were encouraged to maintain the food intake and might not be different from usual during the experiment period.

### Measurement of VO_2peak_

VO_2peak_ was measured during breath-by-breath assessment using an incremental cycle exercise test on a cycle ergometer (MINATO, AE-310SRD, Osaka, Japan). Incremental cycle exercise began at a work rate of 60 W (30–90 W) for men and 30 W (0–60 W) for women, and power output was increased by 15 W•min^−1^ until the subjects could not maintain a fixed pedaling frequency of 60 rpm. The subjects were encouraged during the ergometer test to exercise at maximum intensity. Heart rate and rating of perceived exertion (RPE) were monitored minute by minute during the exercise. RPE was obtained using the modified Borg scale. The highest 30-second averaged value of VO_2_ during the exercise test was designated as VO_2peak_ if three out of four of the following criteria were met: (I) plateau in VO_2_ with an increase in external work, (II) maximal respiratory exchange ratio ≥1.1, (III) maximal heart rate ≥90% of the age-predicted maximum (208–0.7× age; [23]), and (IV) RPE ≥18.

### Measurement of the carotid β-stiffness index

Carotidβ-stiffness was examined as an indicator of arterial stiffness. A combination of ultrasound imaging of the pulsatile common carotid artery and simultaneous applanation of tonometrically obtained arterial pressure from the contralateral carotid artery allowed noninvasive determination of arterial compliance [8]. The carotid artery diameter was measured from images obtained using an ultrasound system equipped with a high-resolution linear array transducer [24]. A longitudinal image of the cephalic portion of the common carotid artery was acquired 1–2 cm proximal to the carotid bulb. All image analyses were performed by the same investigator.

Pressure waveforms and amplitudes were obtained from the common carotid artery using a pencil-shaped probe with a high-fidelity strain gauge transducer (SPT-301; Millar Instruments; Houston, TX; [8]). Because baseline blood pressure levels are dependent on hold-down pressure, the pressure signal obtained via tonometry was calibrated by equating the carotid mean arterial blood pressure and DBP to brachial artery values [8], [24]. The carotid β-stiffness index was calculated using the equation [ln(P1/P0)]/[(D1–D0)/D0], where D1 and D0 are the maximum (systolic) and minimum (diastolic) diameters, and P1 and P0 are the highest (systolic) and lowest (diastolic) blood pressures, respectively [25].

### Measurement of plasma NOx concentrations

NOx concentrations in the plasma were measured by the Griess assay (R&D Systems, Minneapolis, MN), according to the manufacturer's protocol. All samples were assayed in duplicate. Optical density at 540 nm was qualified using a microplate reader (xMark microplate spectrophotometer; Bio-Rad Laboratories, Hercules, CA). Samples were converted into concentration by a linear fit of the log–log plot of the standard curve.

### Measurement of plasma apelin concentrations

Apelin concentrations in the plasma were measured by an enzyme-linked immunosorbent assay (ELISA; Phoenix Pharmaceuticals inc., Burlingame, CA, USA), according to the manufacturer's protocol. Apelin-12, which was used in the present study, has cross-reactivity with apelin-13 and apelin- 36. All samples were assayed in duplicate. Optical density at 450 nm was qualified using a microplate reader (xMark microplate spectrophotometer; Bio-Rad Laboratories). Samples were converted into concentration by a linear fit of the log–log plot of the standard curve.

### Measurements of serum cholesterol and triglyceride levels

Fasting serum concentrations of total cholesterol, HDL cholesterol, and triglyceride levels were determined using standard enzymatic techniques.

### Statistical analysis

Values are expressed as the means ± SE. Differences between groups and two time points were assessed by two-way repeated-measure ANOVA, followed by a Fisher's post hoc test that was applied when a measurement was significantly different. The unpaired Student t-tests were used to compare differences in percent change from baseline in carotid β-stiffness, plasma NOx concentrations, and plasma apelin concentrations between training and control groups. Relationships between plasma apelin concentrations and carotid β-stiffness, plasma NOx concentrations were determined using the Pearson correlation coefficient. *P*<0.05 was defined as statistically significant. All statistical analyses were performed using StatView (5.0, SAS Institute, Tokyo, Japan).

## Results

### A comparison of baseline in the training and control groups

**Figure 1:**
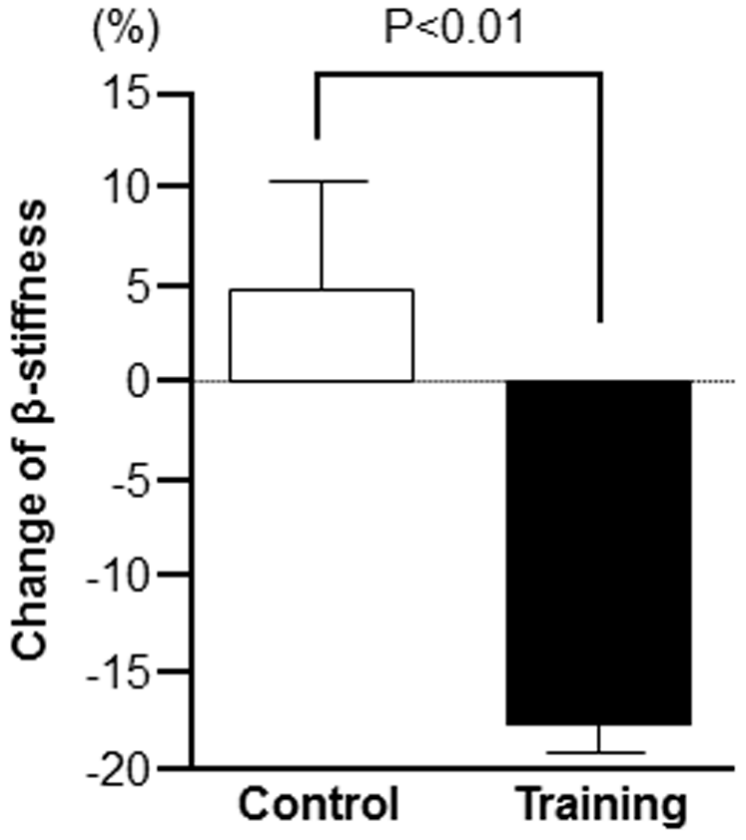
Percent change of carotid β-stiffness in middle-aged and older adults before and after 8 weeks of either exercise training (Training group, *n* = 18) or sedentary living (Control group, *n* = 16). Open bar: sedentary control group, solid bar: aerobic exercise–training group. Data are expressed as the means ± SE.

Before the exercise training intervention, there was no significant difference in VO_2peak_ between the training and control groups, however, there was a significant interaction of groups and time in VO_2peak_ (Table 1, *P* = 0.046). Specifically, in the training group, VO_2peak_ was significantly increased after the exercise training intervention (Table 1). Although there was no significant difference in carotid β-stiffness between the training and control groups before the exercise training, there was a significant interaction between groups and time on carotid β-stiffness (Table 1, *P* = 0.038). After the exercise training intervention, the carotid β-stiffness significantly decreased (Table 1). In addition, the percent change of carotid β-stiffness was also significantly higher in the training group compared to control group (Fig. 1). However, no significant changes were noted between before and after the exercise training intervention, or between groups, in age, height, body weight, BMI, HR, SBP, DBP, total cholesterol levels, HDL cholesterol levels, or triglyceride levels (Table 1).

**Table 1:** Comparison of characteristics in training and control groups.

	Control		Training		Two-way ANCOVA
	Pre	Post	Pre	Post	
Age, years	67.8±1.5	68.1±1.5	66.4±2.1	66.6±2.1	0.981
Height, cm	160.3±2.3	160.0±2.3	159.6±2.2	159.6±2.2	0.997
Body weight, kg	56.4±3.1	56.4±3.1	62.7±2.6	62.5±2.7	0.941
BMI, kg/m^2^	21.8±0.9	21.9±0.9	24.7±1.0	24.6±1.0	0.951
HR, bpm	59.4±2.0	56.6±2.0	58.8±1.3	57.8±2.0	0.596
SBP, mmHg	133.3±3.5	129.3±4.0	129.8±4.2	121.3±3.8	0.531
DBP, mmHg	77.8±2.0	75.5±2.2	77.6±2.8	74.2±2.7	0.824
β-Stiffness, A.U.	15.2±0.8	15.4±0.6	13.7±0.6	11.2±0.5*	0.049
Total cholesterol, mmol/l	5.93±0.24	5.70±0.24	5.48±0.18	5.62±0.20	0.369
HDL cholesterol, mmol/l	2.05±0.16	2.05±0.17	1.77±0.13	1.79±0.14	0.917
Triglycerides, mmol/l	1.24±0.27	1.26±0.31	1.55±0.24	1.37±0.19	0.637
Plasma apelin, ng/ml	2.78±0.18	2.94±0.16	2.31±0.20	5.00±0.39*	0.001
Plasma NOx, μmol/l	21.58±2.00	18.50±2.38	24.46±2.18	51.36±4.35*	0.001
VO_2peak_, ml/kg/min	26.7±1.6	26.5±1.4	23.2±1.3	28.9±1.5*	0.013

BMI: body mass index, SBP: systolic blood pressure, DBP: diastolic blood pressure, HDL: high-density lipoprotein, VO_2peak_: peak oxygen uptake.

Values are means and SE. * P<0.05, after training vs. before training.

Two-way ANOVA was adjusted for sex and age.

### Comparison of plasma apelin and NOx concentrations between the training and control groups

**Figure 2:**
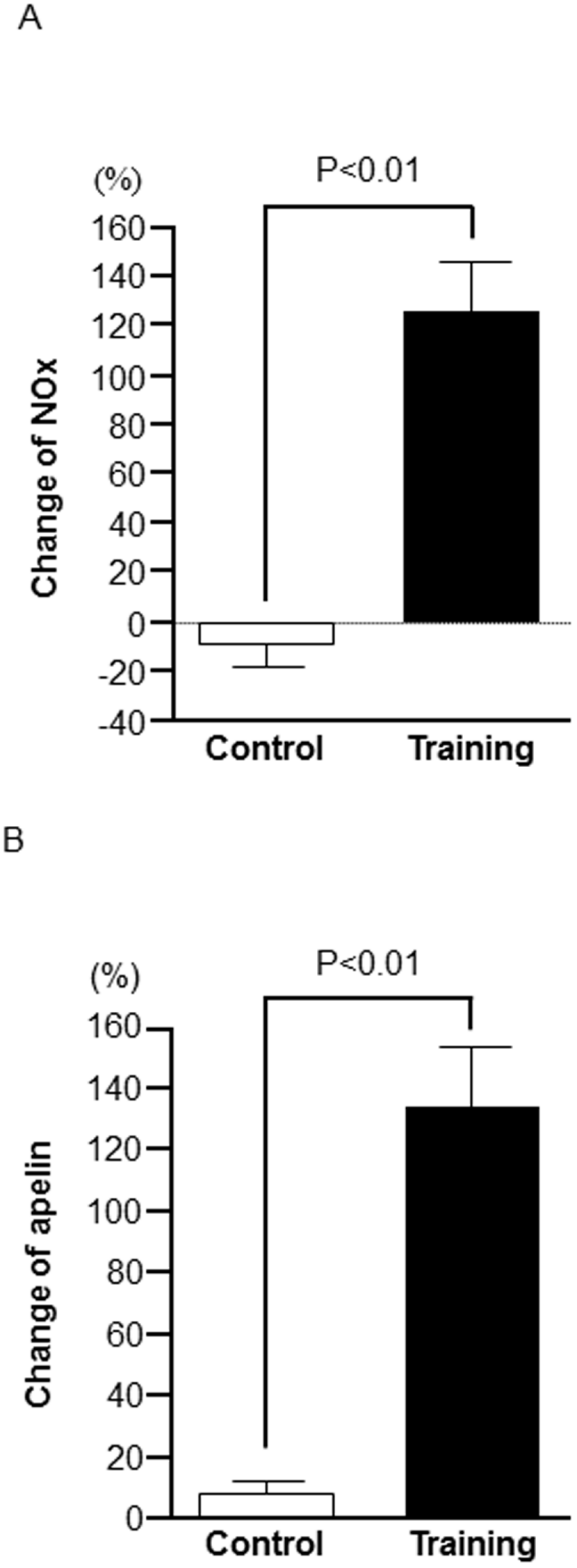
Percent change of plasma nitrite/nitrate (NOx: A) and apelin (apelin: B) concentrations in middle-aged and older adults before and after 8 weeks of either exercise training (Training group, *n* = 18) or sedentary living (Control group, *n* = 16). Open bar: sedentary control group, solid bar: aerobic exercise–training group. Data are expressed as the means ± SE.

Before exercise training intervention, there was no significant difference in plasma apelin and NOx concentrations between the training and control groups. There was a significant interaction between groups and time on plasma NOx concentrations (Table 1, *P*<0.0001). Specifically, after exercise training, plasma NOx concentrations were significantly increased in the training group as compared to the control group (Table 1). Additionally, the percent change in plasma NOx concentrations was significantly higher in the training group than in the control group (Fig. 2-A). A significant interaction among groups and time was seen in plasma apelin concentrations (Table 1, *P*<0.0001). After exercise training intervention, plasma apelin concentrations were significantly increased in the training group as compared to the control group (Table 1). In addition, the percent change of plasma apelin concentrations was also significantly higher in the training group compared to control group (Fig. 2-B).

### The correlation between plasma apelin concentrations and carotid β-stiffness, plasma NOx

**Figure 3:**
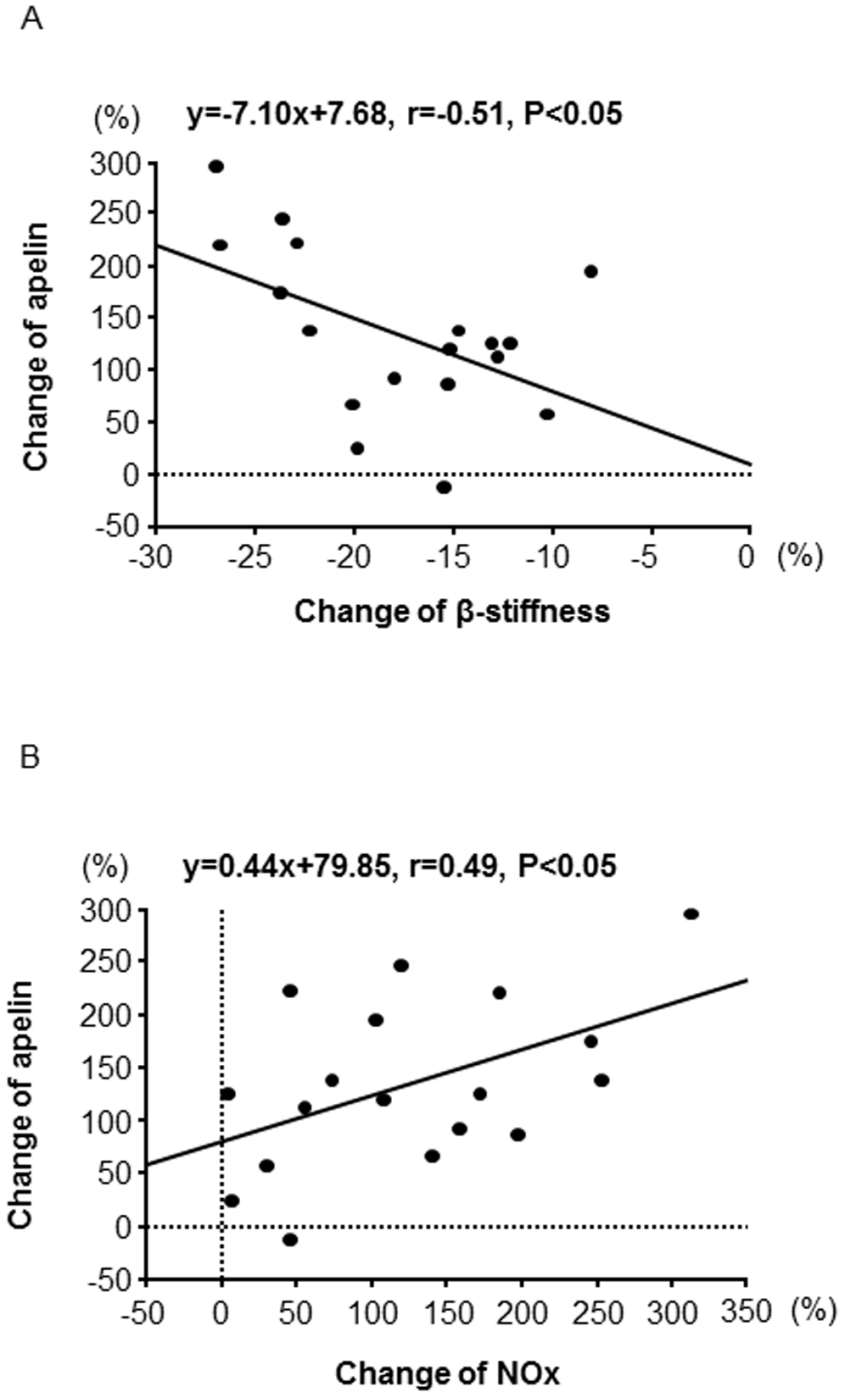
The correlation of percent change between plasma apelin levels and carotid β-stiffness in the training group, which was composed of middle-aged and older adults (A). Correlation between percent change between plasma apelin levels and percent change of plasma NOx concentrations in the training group (B).

In the training group, the percent change of plasma apelin concentrations negatively correlated with the percent change of β-stiffness (r = −0.508, *P* = 0.032, Fig. 3-A). Furthermore, the percent change of plasma apelin concentrations positively correlated with the percent change of plasma NOx concentrations in the training group (r = 0.494, *P* = 0.037, Fig. 3-B).

## Discussion

This study investigated the effects of regular aerobic exercise on apelin concentrations in middle-aged and older adults before and after 8-week aerobic exercise training. After the exercise training intervention, arterial stiffness decreased, concomitantly, plasma apelin levels elevated along with plasma NOx levels. By contrast, there were no significant changes in these parameters in the sedentary control group. Furthermore, the effect of training on carotid β-stiffness was negatively correlated with the effect on plasma apelin levels. Thus, an elevation of plasma apelin levels is associated with a concomitant decrease in arterial stiffness through aerobic exercise training in middle-aged and older adults. Additionally, the effect of training on plasma NOx levels was positively correlated with the effect on plasma apelin levels. Several studies have suggested that increases in NO production resulting from endurance exercise training may be a causal factor in reduction of arterial stiffness risks, in both humans and animals [10], [11], [22]. Therefore, these results suggest that arterial NO bioavailability via apelin may be participated in variation of arterial stiffness in middle-aged and older adults.

In a previous study, circulating apelin levels were increased by 6 months of aerobic training consisting of walking, treadmill running, cycling, or calisthenics at 60–70% of maximal heart rate for 60 min, 4 days/week, whereas 8-types of resistance training at 60–80% of one repetition maximum, 4 days/week, did not change apelin levels in patients with type 2 diabetes mellitus [20]. Patients with higher physical activity had higher plasma apelin levels than less physically physical active patients [21]. In animal studies using hypertensive model rats, 9-week swimming training normalized mRNA expression and tissue concentration of apelin in aorta, along with apelin plasma levels [22]. Previously, however, the effect of regular aerobic exercise on apelin concentration in middle-aged and older adults had remained unclear. This study demonstrated that 8-week aerobic exercise training, consisting of cycling on a leg ergometer at 60–70% VO_2peak_ for 45 min, 3 days/week, elevated plasma apelin levels in middle-aged and older adults. Thus, aerobic exercise training may be an effective way to elevate plasma apelin levels and reduce arterial stiffness in both healthy and at-risk subjects.

This study demonstrated elevated NOx plasma levels and decreased arterial stiffness after aerobic exercise training in middle-aged and older adults. In a previous study, middle-aged and older women who performed aerobic exercise training exhibited elevated plasma NOx levels and reduced blood pressure [11]. Aging leads to increased risk of arterial stiffness; this risk is associated with attenuation of NO generation. However, an animal study revealed that eNOS protein and mRNA expression levels in the aortas of older rats were ameliorated by regular exercise training [10]. In this study, plasma apelin levels were increased by regular exercise training. Moreover, plasma NOx levels were associated with plasma apelin levels. In another study, administration of apelin induced NO production and promoted eNOS mRNA expression in the isolated rat aortic tissue, but did not alter iNOS expression [26]. Furthermore, eNOS phosphorylation of isolated endothelial cells in mice was accelerated by the treatment with apelin [17]. Apelin-induced eNOS activation is mediated by the activation of phosphatidylinositol-3 kinase (PI3K)/Akt signaling pathway in endothelial cells, resulting in increased NO production [27]. Apelin treatment increased eNOS phosphorylation in the aorta, but in isolated endothelial cells from APJ-deficient mice, increased eNOS phosphorylation in response to apelin was inhibited [17]. Additionally, in the left internal mammary artery of patients with stable coronary artery disease, exercise training raised eNOS expression and phosphorylation in association with change of Akt phosphorylation [28]. Apelin is involved in regulation of eNOS gene expression, and contributes to NO production in the endothelial cells of aorta [17], [26]. Thus, the elevation in circulating apelin levels induced by regular exercise training may participate in the acceleration of NO generation in middle-aged and older adults.

We demonstrated that exercise training in middle-aged and older adults elevated apelin plasma concentration. However, the source of the exercise training–induced increase in apelin plasma levels is unclear. Zhang et al. [22] demonstrated that aortic, myocardial, and plasma apelin concentrations were all increased by exercise training in hypertensive rats, concomitant with elevation of the apelin mRNA expression level in aorta and heart. Therefore, aorta and heart tissues are one possible source of the exercise training–induced apelin production. However, apelin has also been detected in several tissues, including white adipose tissue and kidney [12], [13]. Further studies should investigate the source of exercise training–induced increase in apelin plasma levels.

After the aerobic exercise training intervention, plasma apelin levels increased, and concomitantly, plasma NOx levels were increased. The mechanism underlying these effects is unclear. Hypoxic inducible factor 1–α, bone morphogenetic protein receptor 2, insulin, tumor necrosis factor–α, and mechanical stress are all potential inducers of apelin production [27]. However, it is unclear whether the levels of these inducers are influenced by exercise training, and whether they contribute to elevated apelin production in this context. Further studies are needed to examine the effects of regular exercise training on these inducers in endothelial cells. Although the older increase the risk of cardiovascular disease, the present study recruited healthy middle-aged and older subjects. Therefore, it should focus on the effect of exercise training on apelin production in the elderly patient.

In conclusion, we investigated the effects of regular aerobic exercise on plasma apelin concentrations in middle-aged and older adults before and after 8-week aerobic exercise training. After the exercise training intervention, plasma apelin levels increased along with plasma NOx levels, whereas arterial stiffness decreased. Additionally, the plasma apelin level was negatively correlated with carotid β-stiffness, and also was positively correlated with the plasma NOx level. Thus, the increase in plasma apelin levels may partly contribute to the improvement in arterial stiffness and NO bioavailability resulting from aerobic exercise training in middle-aged and older adults.
